# Deletion of the Epidermal Protease *KLK5* Aggravates the Symptoms of Congenital Ichthyosis *CDSN*-nEDD

**DOI:** 10.3390/ijms26178605

**Published:** 2025-09-04

**Authors:** Eleni Zingkou, Marie Reynier, Georgios Pampalakis, Guy Serre, Nathalie Jonca, Georgia Sotiropoulou

**Affiliations:** 1 Department of Pharmacy, School of Health Sciences, University of Patras, Rion, 26504 Patras, Greece; gpampalakis@pharm.auth.gr (G.P.); gdsotiro@upatras.gr (G.S.); 2 Toulouse Institute for Infectious and Inflammatory Diseases (INFINITy), CNRS, Toulouse University, Inserm, 31024 Toulouse, France; marie.reynier1@gmail.com (M.R.); guy.serre@udear.cnrs.fr (G.S.); 3 Laboratory of Pharmacology, School of Pharmacy, Aristotle University of Thessaloniki, 54124 Thessaloniki, Greece; 4 Department of Cell Biology and Cytology, Federative Institute of Biology, Purpan University Hospital, 31300 Toulouse, France

**Keywords:** *CDSN*-nEDD (Peeling skin syndrome type 1), Epidermal Differentiation Disorders (EDD), kallikrein 5 (KLK5), corneodesmosin (CDSN), human epidermal equivalent (HEE), mouse models, epidermal proteolysis, epidermal lipids

## Abstract

Congenital ichthyoses, now grouped under the acronym EDD (Epidermal Differentiation Disorders), include nonsyndromic forms (nEDD) that may be caused by loss-of-function mutations in the *CDSN* gene encoding corneodesmosin (*CDSN*-nEDD, formerly Peeling skin syndrome type 1). It is characterized by skin peeling, inflammation, itching and food allergies, while no specific therapy is currently available. High levels of KLK5, the serine protease that initiates the desquamation cascade, are found in the epidermis of *CDSN*-nEDD patients. Thus, we hypothesized that KLK5 inhibition would alleviate the symptoms of *CDSN*-nEDD and could serve as a new pharmacological target. A human epidermal equivalent (HEE) model for *CDSN*-nEDD was developed using shRNA-mediated *CDSN* knockdown. This model was characterized and used to assess the role of KLK5 knockdown on *CDSN*-nEDD. Also, *Klk5^−/−^* mice were crossed with *Cdsn^epi−/−^* mice, the murine model of *CDSN*-nEDD, to examine in vivo the effect(s) of *Klk5* deletion in *CDSN*-nEDD. Both models recapitulated the *CDSN*-nEDD desquamating phenotype. Elimination of KLK5 aggravated the *CDSN*-nEDD phenotype. Epidermal proteolysis was surprisingly elevated, while severe ultrastructural (corneo)desmosomal alterations increased epidermal barrier permeability and *stratum corneum* detachment was manifested. Based on these results, we concluded that targeting epidermal proteolysis with *KLK5* ablation cannot compensate for the loss of corneodesmosin and rescue over-desquamation of the *CDSN*-nEDD. Possibly, in the absence of KLK5, other proteases take over which increases the severity of over-desquamation in *CDSN-nEDD*. The translational outcome is that over-desquamation may not always be rescued by eliminating epidermal proteolysis, but fine protease modulation is more likely required.

## 1. Introduction

Corneodesmosin-nonsyndromic Epidermal Differentiation Disorder [*CDSN*-nEDD (formerly Peeling skin syndrome type 1, OMIM270300)] [1] is a very rare autosomal recessive form of congenital ichthyosis. It was identified using a whole genome analysis approach in a large family of consanguineous Roma in Germany, where four members showed superficial skin peeling from birth [2]. Since then, only a few cases have been reported worldwide, with the incidence of the disease estimated at 0.11 per million in France [3]. It is caused by homozygous inactivating mutations in *CDSN* gene encoding corneodesmosin (CDSN). This protein forms specialized junctions known as corneodesmosomes, which hold together the corneocytes in the *stratum corneum* (SC). The disease is characterized by skin over-desquamation, inflammation, food allergies and itching that significantly affect their quality of life, albeit their life expectancy is normal [2,4]. These symptoms highly resemble the symptoms of *SPINK5*-sEDD (formerly Netherton syndrome, OMIM 256500). Even trichorrhexis invaginata (bamboo hair), a feature considered pathognomonic of *SPINK5*-sEDD, has been reported in a *CDSN*-nEDD patient [5]. As with other forms of ichthyoses, no effective treatment of *CDSN*-nEDD is currently available. Thus, patients receive only symptomatic therapy (e.g., emollients, antibiotics, steroids) to alleviate disease symptoms. Global or epidermal conditional *Cdsn^−/−^* mice recapitulate the disease phenotype and as in many cases of invalidation of genes expressed in late differentiation of epidermis, these mice die soon after birth [6,7].

Due to the phenotypic similarities between *CDSN*-nEDD and *SPINK5*-sEDD, diagnosis can only be succeeded with immunohistochemical staining demonstrating the absence of CDSN or LEKTI, the product of the *SPINK5* gene, respectively. Definitive diagnosis requires DNA sequencing of the respective genes. The complexity for diagnosing ichthyoses was highlighted previously [8,9].

Epidermal desquamation depends on the tightly regulated activity of the epidermal kallikrein-related peptidases (KLKs). It is well-established that the KLK5, KLK7 and KLK14 members of the KLK family cleave the corneodesmosomal proteins, namely desmoglein 1, desmocollin 1, and CDSN. The process is controlled by the endogenous inhibitor LEKTI which quenches their activities through the formation of KLK-LEKTI complexes. These complexes dissociate only at the outermost SC layers that has acidic pH due to the acidic mantle. Therefore, only the outer layers of SC are shed [10,11,12]. KLK5 acts as the upstream activator of the epidermal proteolytic cascade, since it can autoactivate and, subsequently, activate the other KLK pro-enzymes, thus augmenting the proteolytic activities in the epidermis [10].

*SPINK5*-sEDD is due to inactivating mutations in the *SPINK5* gene encoding for LEKTI. Thus, in the absence of LEKTI, the KLKs are activated at the *stratum granulosum* (SG)-SC boundary and allow for premature shedding of SC [12]. Accordingly, the elimination of the *Klk5* gene in *Spink5^−/−^* mice, the *SPINK5*-sEDD murine model, rescued neonatal lethality [13]. To this end, it should be emphasized that inhibitors for KLK5 are under development [14,15,16,17,18,19]. A borolane-based inhibitor known as GSK951 exhibiting increased selectivity and low IC50 of 250 pM, showed therapeutic efficacy in the transgenic KLK5 mouse model of *SPINK5*-sEDD [20]. These inhibitors could also be important for the treatment of other more common diseases governed by upregulated KLK5, such as rosacea [21] and atopic dermatitis [22]. Finally, a bispecific KLK5/KLK7 antibody has been generated and tested in the mouse model of *SPINK5*-sEDD and atopic dermatitis [23]. If KLK5 proves to be the main player in other skin diseases as in *CDSN*-nEDD, this will expand the disease repertoire where these compounds can be administered, while exerting pressure for their clinical implementation.

In contrast, in *CDSN*-nEDD, over-desquamation is due to the defective structure of corneodesmosomes. Nevertheless, in *CDSN*-nEDD, a strong elevation of the KLK5 expression was found [2,24]. In addition, it has been suggested that CDSN protects corneodesmosomes from premature proteolysis [25]. Therefore, we hypothesized that this abnormally high KLK5 expression in combination with the absence of CDSN expression could lead to enhanced cleavage of corneodesmosomes, and to over-desquamation. Further support for the rationale of targeting KLK5 for the treatment of *CDSN*-nEDD comes from the observation that absence of *Klk5* results in increased number and length of desmosomes [26]. To investigate whether KLK5 targeting could attenuate the symptoms of *CDSN*-nEDD, we employed two different models: (a) an in vitro organotypic culture and (b) an in vivo mouse model. The in vitro model consisted of a human epidermal equivalent (HEE) model where the genes *KLK5* and *CDSN* were co-knocked down by shRNA interference, while the in vivo model involved the generation and study of double knockout *Cdsn^epi−/−^Klk5^−/−^* mice. Contrary to our original hypothesis, KLK5 exacerbated the *CDSN*-nEDD phenotype.

## 2. Results

### 2.1. Production and Validation of the HEE Model for CDSN-nEDD

Single and double gene expression knockdown was achieved using shRNA in normal human primary keratinocytes, which were secondarily used to generate HEE. Efficiency of single and double gene silencing was controlled at the RNA and protein level by RT-qPCR and Western blot, respectively (Figure S1). *CDSN* and/or *KLK5* mRNA expressions were always decreased by 65% or more in comparison to the corresponding control (shCtrl puro, shCtrl neo or shCtrl puro-neo).

### 2.2. KLK5 Gene Silencing Exacerbates the Histological and Ultrastructural Abnormalities Observed in shCDSN HEE

**Figure 1:**
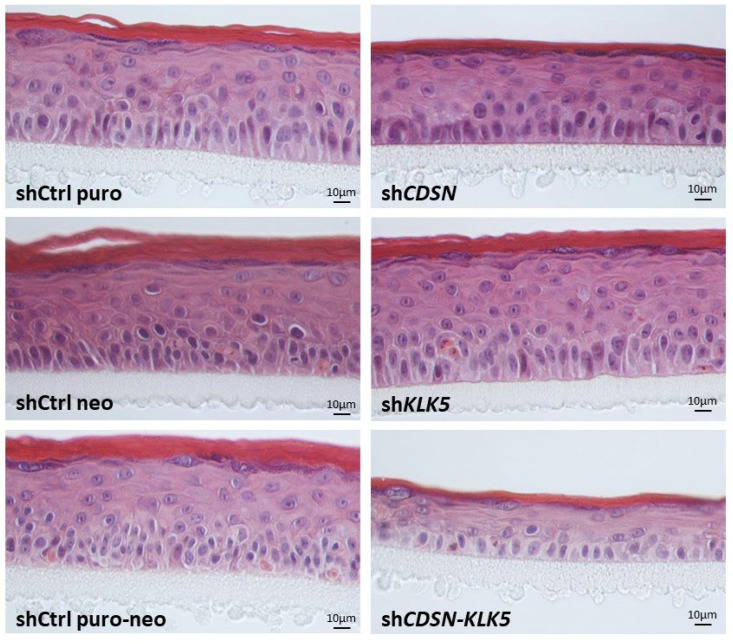
Histological analysis of HEEs produced with sh*CDSN* and/or sh*KLK5* keratinocytes. The HEEs generated from sh*CDSN* keratinocytes showed thinner epidermis compared to the controls or the sh*KLK5* keratinocytes-derived HEEs. Notably, co-silencing of *CDSN* and *KLK5* further reduced the thickness of the HEEs. Representative images of paraffin-embedded HEE sections observed after hematoxylin-eosin staining are shown (n ≥ 4 HEE/condition).

HEEs with single or double gene silencing were fully differentiated within 12 days of culture at the air–liquid interface, as it was the case with control shRNA HEEs. Control and sh*KLK5* HEE had a similar morphology, whereas the global thickness of sh*CDSN* HEE (living layers and SC) was reduced. This effect appeared amplified in the case of the double sh*CDSN*-*KLK5* silencing (Figure 1). Observation of sh*CDSN* HEE by TEM confirmed the histological observations and the detachment between the SG–SC boundary, which never occurred in the control HEE.

**Figure 2:**
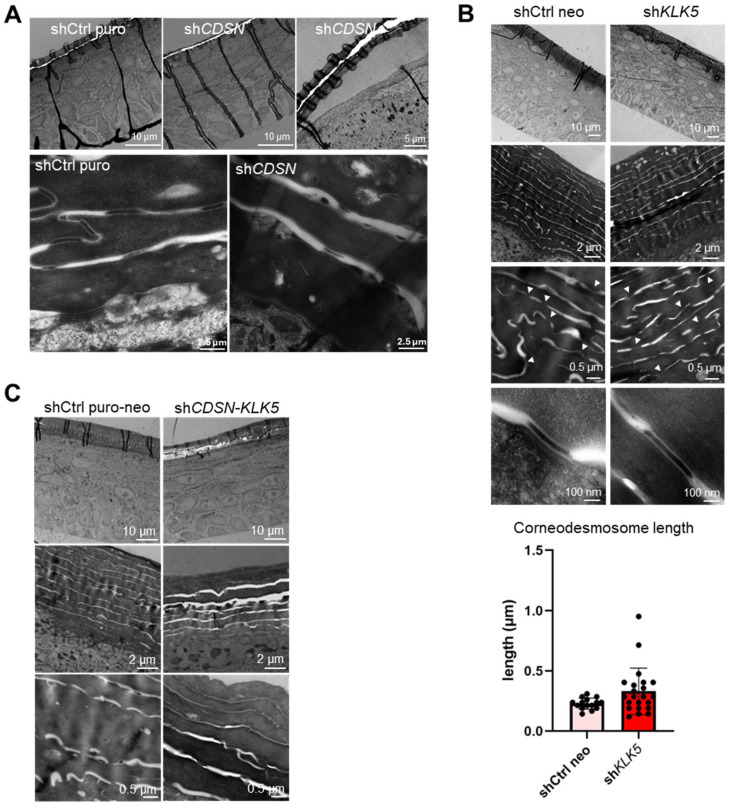
Ultrastructural analysis of the HEEs generated with sh*CDSN* and/or sh*KLK5* keratinocytes. Representative images of HEE sections observed by transmission electron microscopy (TEM) are shown for sh*CDSN*: (**A**) sh*KLK5* (**B**) and sh*CDSN*-*KLK5* (**C**). White arrowheads indicate the corneodesmosomes. In the SC of shCtrl neo and sh*KLK5* HEE, the length of corneodesmosomes was measured (n = 15 and 22 corneodesmosomes, respectively) and shown in (**B**). Importantly, the worsening of the HEE structural phenotype obtained from co-silencing *CDSN* and *KLK5* in keratinocytes was observed. Specifically, the SC was thinner, loosely cohesive, with enlarged intercorneocyte spaces and aberrant corneodesmosomes. (n ≥ 2 HEEs/condition).

Moreover, *CDSN* depletion led to a slight widening of the intercorneocyte spaces and corneodesmosomes appeared smaller and less cohesive (Figure 2A). The general structure and the morphology of the SC of sh*KLK5* HEE was like control HEE. However, *KLK5* silencing had an impact on the number of corneodesmosomes as well as their length which increases (although with high variability, *p* = 0.076) in comparison to shCtrl Neo-HEE (Figure 2B). This is in accordance with the significantly increased number of desmosomes in the epidermis of *Klk5^−/−^* mice relative to wt [26]. Finally, ultrastructural observation of sh*CDSN*-*KLK5* HEE revealed a phenotype similar to sh*CDSN* but exacerbated. In particular, the SC was thinner, loosely cohesive, with enlarged intercorneocyte spaces and aberrant corneodesmosomes (Figure 2C).

### 2.3. KLK5 Gene Silencing Worsens the Impaired Permeability Barrier Observed in the shCDSN HEEs

**Figure 3:**
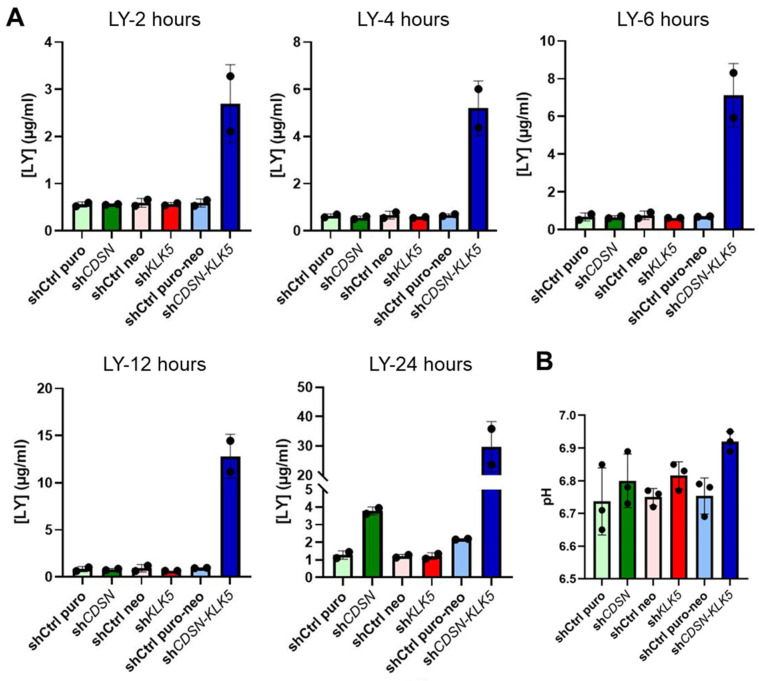
Effect of *CDSN* and/or *KLK5* gene silencing on the epidermal barrier function and the SC pH of HEEs. (**A**) Lucifer yellow (LY) dye penetration assay. A LY solution was applied on the surface of HEE and the dye concentration in the culture medium was measured fluorometrically in a fluoroscan at the indicated times (n = 2 independent experiments for each condition). (**B**) Measurement of pH of the external surface of the indicated HEEs (n = 3 independent experiments for each condition).

The permeability barrier, surface pH and the SC lipids were determined for HEEs. The effect of *CDSN* and/or *KLK5* silencing on the permeability barrier function of HEE was assessed by dye exclusion (lucifer yellow) assay. After 24 h, sh*CDSN* HEE showed an elevated permeability defect in comparison to control (shCtrl puro). *KLK5* silencing alone had no effect on the outside–in permeability barrier. In contrast, when both *CDSN* and *KLK5* gene expression were inhibited, a barrier defect was detected within the first hours of incubation and dramatically increased with time. Twenty-four hours after dye application, its concentration in the culture medium was around thirty-times higher in the case of sh*CDSN*-*KLK5* HEE than in controls or single gene knockdown HEE (Figure 3A). The pH at the surface of the SC of sh*CDSN*-*KLK5* HEE was not significantly different in comparison with shCtrl puro-neo HEE (6.92 vs. 6.75, mean values) (Figure 3B).

Since lipid organization in the SC is important for the normal function of the epidermal barrier, we analyzed the extracellular lipids in the SC of the various HEEs by oil red O staining. We did not observe any effect of *CDSN* and/or *KLK5* gene silencing on the staining of neutral lipids in the SC (Figure S2A). During cornification, some lipids are covalently bound to the protein shell of the cornified envelopes to form mature hydrophobic envelopes. We assessed cornified envelope maturation by nile red/involucrin double staining of envelopes purified from control HEEs, *CDSN* and/or *KLK5* knockdown HEEs. We found mature hydrophobic envelopes as well as immature involucrin-positive envelopes in similar proportions in each HEE condition (Figure S2B).

### 2.4. Deletion of Klk5 in Cdsn^epi−/−^ Mice Does Not Improve the Phenotype of CSDN-nEDD

The *Cdsn^epi−/−^* died within 1 h from birth due to severe desquamation that led to extensive dehydration [6]. Here, the *Cdsn^epi−/−^Klk5^−/−^* (DKO) mice were generated according to the crossing scheme depicted in Figure S3.

**Figure 4:**
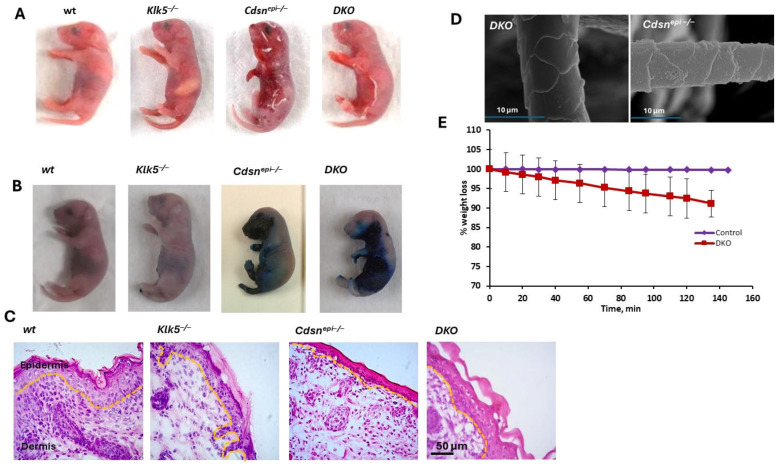
Deletion of the *Klk5* gene in *Cdsn^epi−/−^* background does not rescue neonatal lethality. (**A**) Macroscopic appearance of neonatal DKO mice shows severe signs of desquamation. (**B**) Toluidine blue dye penetration assay shows that DKO and *Cdsn^epi−/−^* mice exhibit severe epidermal barrier defect. (**C**) HE staining of skin sections reveal complete absence of SC in the *Cdsn^epi−/−^* sample and complete detachment in the DKO sample. (**D**) No morphological alterations in the whisker structure of DKO and *Cdsn^epi−/−^* are shown. (**E**) DKO mice exhibit rapid weight loss due to dehydration.

The DKOs appeared normal at birth, but within a few minutes from birth, DKOs developed a dry and wrinkled appearance accompanied with SC detachment and the development of a red, shiny appearance, uniformly resulting in mice death ~7 h after birth. This phenotype is reminiscent of *Cdsn^epi−/−^* neonates phenotype (Figure 4A). The deterioration of epidermal barrier was demonstrated with toluidine blue dye exclusion assay (Figure 4B). Histology examination of skin sections from neonatal *Cdsn^−/−^Klk5^−/−^* mice revealed persistent SC detachment suggesting that elimination of *Klk5* is not sufficient to ameliorate the cutaneous hallmarks of *CDSN*-nEDD (Figure 4C). The weight of DKO is normal at birth but is rapidly reduced due to dehydration (Figure 4D). Examination of the whisker morphology with SEM did not show any abnormalities (Figure 4E). Therefore, deletion of *Klk5^−/−^* does not appear to rescue the lethal over-desquamating phenotype of *Cdsn^−/−^* mice.

### 2.5. DKO Mice Display Ultrastructural Alteration Similar to Those Observed in shCDSN-KLK5 HEEs

**Figure 5:**
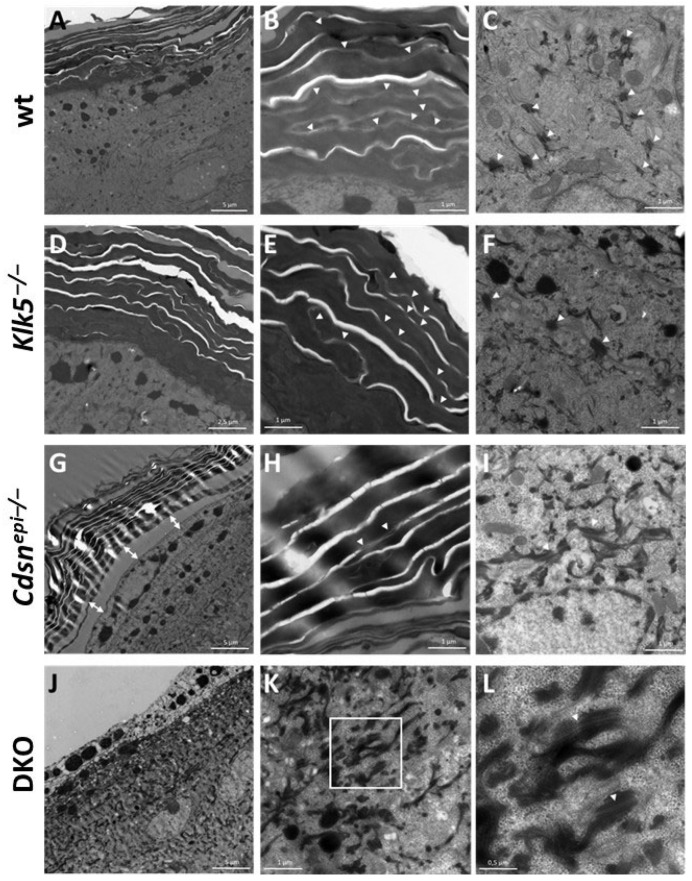
Representative TEM images of the epidermis of wt (**A**–**C**), *Klk5^−/−^* (**D**–**F**), *Cdsn^epi−/−^* (**G**–**I**) and *DKO* (**J**–**L**) mice. (**A**,**D**,**G**,**J**): Upper epidermis; (**B**,**E**,**H**): higher magnification in the SG; (**C**,**F**,**I**,**K**,**L**): SG. White arrowheads show corneodesmosomes in (**B**,**E**,**H**) and desmosomes in (**C**,**F**,**J**,**L**). Double white arrows in (**G**) show abnormal spacing between the SG and the SC in *Cdsn^−/−^* epidermis. Note the total absence of SC in *DKO* epidermis. (**L**) corresponds to a magnification of the square in (**K**). Bar = 5 µm (**A**,**G**,**J**), 2.5 µm (**D**), 1 µm (**B**,**C**,**E**,**F**,**H**,**I**,**K**) and 0.5 µm (**L**).

At the ultrastructural level, *Klk5^−/−^* epidermis appeared quite like the wt epidermis, with numerous desmosomes in the SG and corneodesmosomes in the SC (Figure 5A–F). In *Cdsn^−/−^* epidermis, the SC was loosely cohesive and abnormal widening of the SG/SC interface was often observed (Figure 5G). There were less numerous corneodesmosomes in the SC, whereas desmosomes with normal appearance were present in the SG (Figure 5G–I). The DKO epidermis showed a more drastic phenotype. The SC was totally absent from the observed samples, most probably it detached from the skin surface because of lack of cohesion. Consequently, the underlying SG appeared damaged, but a few desmosomes could be observed in this epidermal layer (Figure 5J–L).

### 2.6. Aberrant Overall Proteolytic Activities in DKO Epidermis

**Figure 6:**
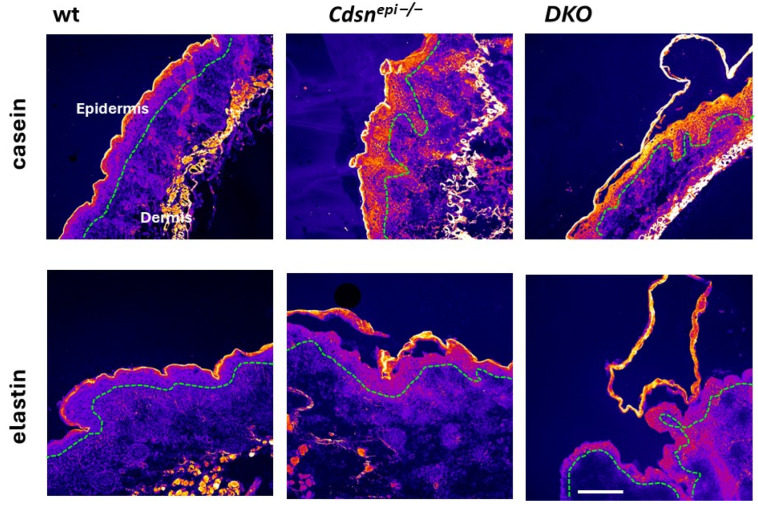
Elevated epidermal proteolytic activities in *Cdsn^−/−^* and *DKO* epidermis. In situ zymography using fluorescence-quenched elastin or casein. Skin tissue sections were prepared from wt, *Cdsn^epi−/−^* and *Cdsn^epi−/−^Klk5^−/−^* (DKO) mice. Fluorescence intensity data was transformed into a color gradient (as shown) using ImageJ software (1.52a). In situ zymography FL substrates (casein, and elastin) show elevated overall epidermal proteolysis in *Cdsn^epi−/−^* and *DKO* neonates. The green dashed line indicates the epidermis–dermis junction. Scale bar = 50 µm.

Since KLK5 is the initiator of the epidermal KLK cascade, we examined whether elimination of *Klk5* could have a limiting effect on epidermal proteolytic activities in the DKO model. For this, in situ zymography using casein and elastin quenched fluorescent substrates was used. Interestingly, the epidermis of *Cdsn^epi−/−^* and DKO mice exhibited highly increased proteolytic activities. Therefore, deletion of *Klk5* cannot reduce the increased overall epidermal proteolysis in *Cdsn^−/−^* epidermis (Figure 6).

**Figure 7:**
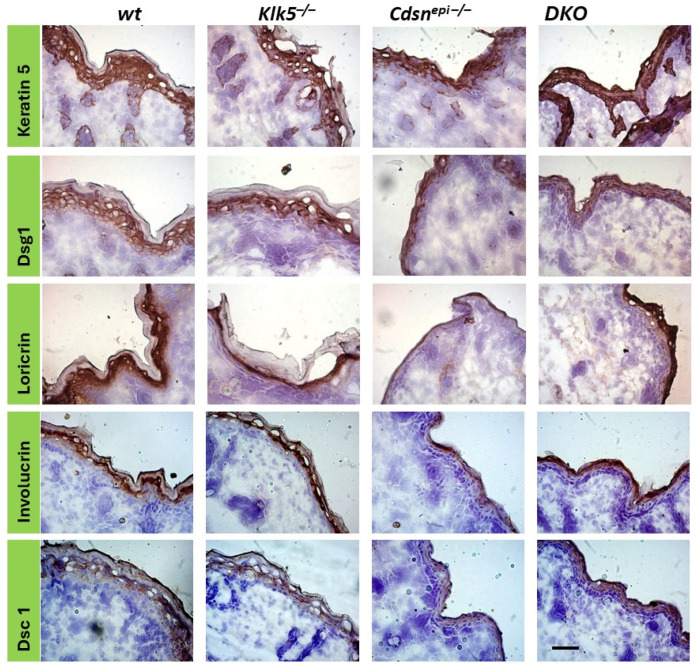
Normal differentiation process and normal expression of Dsg1 and Dsc1 in DKO epidermis. The expression of keratin 5, Dsg1, loricrin, involucrin and Dsc1 was analyzed with immunohistochemistry (IHC). No significant changes were found in *Cdsn*-deficient and *Cdsn^−/−^Klk5^−/−^* epidermis. Scale bar = 50 µm.

Then, we studied the expression of characteristic molecules of epidermal differentiation in *Cdsn^epi−/−^* and DKO epidermis. As shown in Figure 7, no changes in the expression pattern relative to the controls were found. This indicates that epidermal differentiation is not affected by deletion of *Cdsn* or *Cdsn* and *Klk5*. The normal differentiation pattern in *Cdsn^epi−/−^* epidermis is in accordance with a previous study [6].

### 2.7. Lipid Characterization of the DKO Epidermis and Human SC Sample

**Figure 8:**
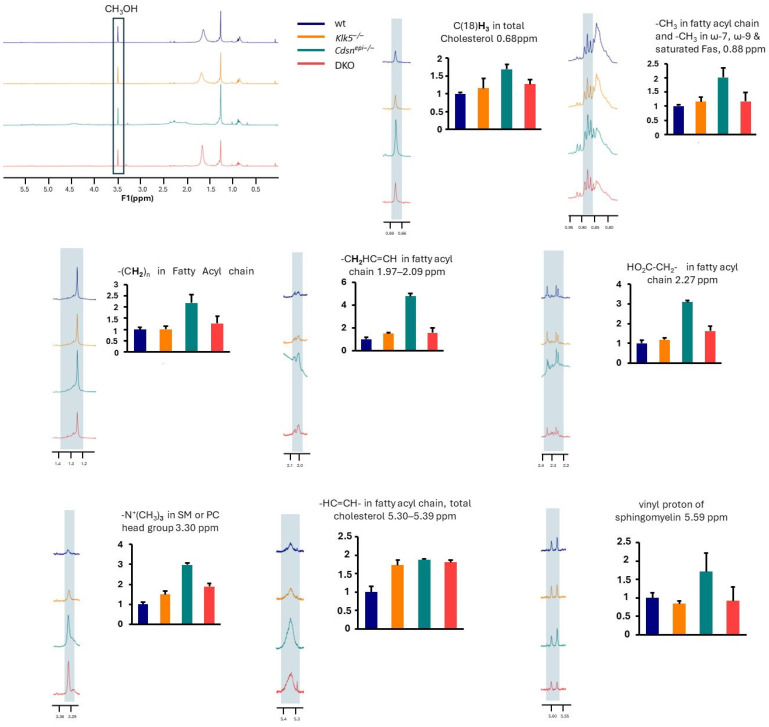
Quantification of various lipid classes in the epidermis of mouse models. The ^1^H-NMR peaks associated with different lipid structures at certain chemical shifts (in ppm) are shown at the right of each graph and under the same magnification. The area under the peak was integrated and compared using the MestReNova software (https://mestrelab.com/). The epidermal lipid profile appears to be normalized after deleting *Klk5* in *Cdsn^epi−/−^*. The upper left picture shows the representative ^1^H-NMR spectra, while the other spectra used for quantification of certain lipid classes represent a magnification of the corresponding region (n = 4 for *Cdsn^epi−/−^Klk5^−/−^* and n = 3 for all other genotypes).

The lipid profile was analyzed with ^1^H-NMR and the results are shown in Figure 8. Significant differences in the epidermal lipidome between control mice including wt and *Klk5^−/−^* and disease mouse models *Cdsn^epi−/−^* and *DKO* were observed. Total cholesterol is upregulated in *Cdsn^epi−/−^* epidermis while it is almost normalized in *DKO* epidermis. Quantification of the peaks corresponding to -N^+^(CH_3_)_3,_ which is present in sphingomyelin (SM) or phosphatidylcholine (PC), and to vinyl group with unique resonance at 5.6 ppm, which is present in SM, shows that these lipids are increased *in Cdsn^epi−/−^* while they are normalized in *DKO*. In addition, the lipids in the *Klk5^−/−^* epidermal extracts are higher than in wt. The alteration of lipid profiling in *Cdsn^epi−/−^* upon deletion of KLK5 suggests that the production of epidermal lipids is regulated by KLK5 either directly or indirectly. Interestingly, the normalization of certain epidermal lipids by deletion of KLK5 does not suffice to restore the epidermal barrier.

**Figure 9:**
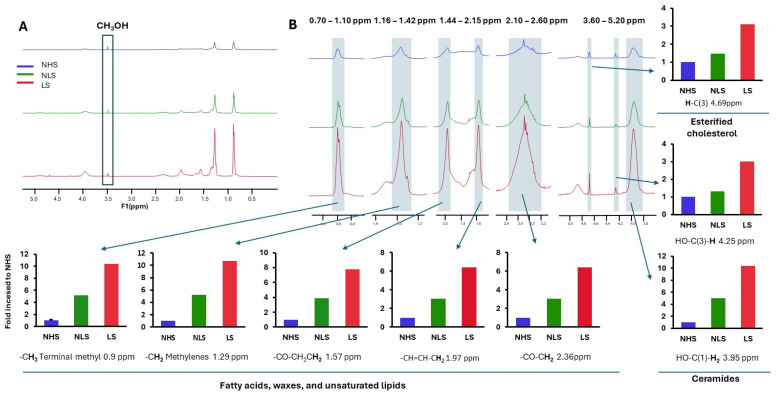
Lipid profile of clinical specimens. (**A**) ^1^H-NMR spectra from a representative lipid extract obtained from the SC of healthy individual (NHS), a non-lesional (NLS) and a lesional site (LS) from a *CDSN*-nEDD patient. The methanol peak used as an internal control for quantification is boxed. (**B**) Magnification of certain regions of the ^1^H-NMR spectrum and their quantification. A strong increase in the amount of fatty acid acyl chains and ceramides is found in the lesional SC skin of *CDSN*-nEDD patient. Further, increased amount of unsaturated lipids is found in the SC of NLS and LS of the *CDSN*-nEDD patient.

Then, we analyzed extracts from SC from two healthy donors and from a lesional and a non-lesional site obtained from a *CDSN*-nEDD patient. Quantification of the peaks corresponding to -CH_3_ terminal methyls, -CH_2_ methylenes, -CH=CH-CH_2_ (vinyl protons indicating high degree of unsaturation) and -CO-CH_3_, which are present in all fatty acyl chains, showed a remarkable increase in the lipid content of lesional *CDSN*-nEDD SC compared to non-lesional skin and normal SC (Figure 9). This resembles the *Cdsn^epi−/−^*. Further, high levels of ceramides and esterified cholesterol were found in the lesional skin compared to unlesional or normal skin.

Fatty acids are important for preserving the epidermal barrier [27]; thus, their increase in the patient sample and in the mouse model may reflect a compensatory mechanism. On the other hand, the increase in unsaturated fatty acid content may be associated with the defects in epidermal barrier. Previously, increased epidermal levels of unsaturated fatty acids as found in atopic dermatitis and *SPINK5*-sEDD were associated with defects in epidermal barrier due to reduction in packing density [28]. Another important factor that regulates the epidermal barrier is the length of carbon chain in fatty acids and ceramides. Decreased carbon length is associated with barrier defect [29]. Here, we have not studied this parameter since such information cannot be extracted from ^1^H-NMR data and will require future mass spectrometry-based approaches for lipid speciation.

No significant alterations in the lipid profile between the control and the *CDSN*-silenced HEEs were found (Figure S2). However, these analyses were conducted using semi-quantitative histological staining techniques, which are significantly less sensitive and precise than ^1^H-NMR. Moreover, oil red O only stains the neutral lipids, and the Nile red staining analysis [30] was not performed on HEE section but on cornified envelopes purified from HEE SC. In contrast, ^1^H-NMR provides analyses of all extractable lipids. Thus, these results cannot be directly compared.

## 3. Discussion

*CDSN*-nEDD is a rare skin peeling disease for which there is no specific therapy. KLK5 is the key enzyme that regulates skin desquamation due to its ability to autoactivate and then activate other KLKs. Further, KLK5 cleaves components of (corneo)desmosomes [10,11]. High levels of KLK5 expression were previously found in *CDSN*-nEDD [2,24]. Here, we used an in vitro HEE and an in vivo model to assess the role of KLK5 in *CDSN*-nEDD. HEEs are valuable models to study rare skin diseases and test potential new therapeutic strategies. In this direction, a skin equivalent (encompassing both epidermis and dermis) for *SPINK5*-sEDD (Netherton syndrome) was previously designed, to test the role of *KLK7* and *KLK5* gene silencing [31], or their chemical inhibition [32] in reversing the disease phenotype. Recently, a protein replacement therapy was suggested for *CDSN*-nEDD after demonstrating that application of recombinant CDSN in liposomes to a *CDSN*-nEDD HEE model improved barrier function and SC structure [33]. However, this strategy will take time to enter clinical practice. Thus, in an attempt to identify new pathways that mediate pathology and that can be targeted for pharmacological manipulation, we examined the role of KLK5 in *CDSN*-nEDD.

When the expression of both *CDSN* and *KLK5* was downregulated in HEE, the cohesive defect at the SG/SC interface was no longer observed, whereas the SC still appeared loosely cohesive and the overall HEE thickness was more importantly reduced in comparison to sh*CDSN* HEE. Moreover, sh*CDSN*-*KLK5* HEE presented an important permeability barrier defect, in comparison to control or single gene knockdown HEE. Thus, the downregulation of both *CDSN* and *KLK5* expressions in HEE resulted in the more dramatic phenotype we observed. On the other hand, *KLK5* silencing in HEE led to a phenotype very similar to that of control HEE, with no impact on either morphology or permeability barrier function. The number and length of (corneo)desmosomes in the sh*KLK5*-HEE model were increased in comparison to control conditions, in accordance with the previously described phenotype of *Klk5^−/−^* mice [26].

Deletion of Klk5 did not rescue the lethal neonatal phenotype of *Cdsn^epi−/−^* mice. The DKO mice exhibited increased epidermal proteolytic activity and severe desquamation leading to dehydration and neonatal lethality. The detachment of SC from the underlying SG was so severe that the SC was lost during preparation for TEM study. Here, both the HEE model and the animals show the same phenotype and defects. Notably, this is not always the case. In the organotypic skin culture model for *SPINK5*-sEDD, silencing of *KLK7* showed a more potent effect in alleviating the phenotype than the *KLK5* silencing. However, later in vivo studies showed that targeting *Klk5* can rescue the neonatal lethality of *Spink5^−/−^* mice, the murine *SPINK5*-sEDD model [13,34], while *Klk7* cannot [34].

Our study suggests that KLK5 does not contribute to the overall epidermal proteolysis in *CDSN*-nEDD and the reported upregulated KLK5 expression may not correlate with activity. However, it should be mentioned that in another study the expression of KLK5 was found reduced in some *CDSN*-nEDD patients, indicating high variability in its expression [35]. Thus, other proteolytic activities could play a role in *CDSN*-nEDD. In this direction, *Klk13* was found highly upregulated in the epidermis of *Cdsn^iepi−/−^* mice [35]. It should be noted that KLK13, KLK6 and KLK14 are implicated in other over-desquamating diseases related to *S. aureus* infections [36]. Thus, KLK13 may be involved in *CDSN*-nEDD, and indeed KLK13, but also KLK7 were found highly upregulated in the skin extract from a *CDSN*-nEDD [35]. In addition, another study found very high levels of KLK6, KLK7, KLK8, KLK11, KLK13, and KLK14 in SC extracts from two *CDSN-nEDD* patients. These expressions were associated with increased SC trypsin-like activities, but not chymotrypsin-like activities (i.e., KLK7) [24]. Plasmin-like activities were also upregulated in the SC extracts [24]. Future studies using new proteomic or degradomic approaches [37] will assist in the delineation of the role of other proteases that take over in the absence of KLK5 in *CDSN*-nEDD. These omic approaches will also allow for the studying of the expression of the major epidermal protease inhibitors in *CDSN*-nEDD, such as SCALP, SLPI and LEKTI [38]. Finally, the potential participation of MMPs or elastase-2 in the phenotype of *CDSN*-nEDD cannot be excluded, especially due to their interplay with KLKs [39].

No change in the differentiation status was found in *Cdsn^epi−/−^* mice, as previously described [6]. However, it may require more time to develop alterations in the differentiation status. In this direction, when the skin of *Cdsn^−/−^* was grafted in nude mice, to study disease progression to adulthood, it was shown that *Cdsn^−/−^* had signs of acanthosis, hyperkeratosis, parakeratosis and Stat3 nuclear localization, resembling a psoriatic phenotype [6]. This observation is consistent with the fact that the gene for *CDSN* is located within the psoriasis susceptibility locus [4].

The *Cdsn^epi−/−^* mice appear to recapitulate the alterations in the lipid profile observed in patients. This should be confirmed in the future since here we only analyzed a SC from one *CDSN*-nEDD patient and compared it with the epidermal extract of lipids from the *Cdsn^epi−/−^*. The reason of this was two-fold; first, SC is much easier to be obtained from *CDSN*-nEDD patients (they desquamate every day) without any invasion, i.e., biopsy; and second, one patient sample was available due to the extreme rarity of the disease. Future studies aiming for the delineation of the epidermal or SC lipidome in a larger cohort of patients is required for more accurate conclusions. Analysis of KLKs expressions or activities in these samples may also aid in the stratification of samples (e.g., high activity vs. low activity) and their correlation with lipid profiling. KLK7 was previously linked with epidermal lipid processing since it was able to cleave β-glucocerebrosidase and acidic sphingomyelinase [40]. Thus, potentially high KLK7 expression, as observed in a patient with *CDSN*-nEDD [35], could lead to reduced sphingomyelinase that translates to increased sphingomyelin content as observed here.

Overall, both of our models, namely the HEE and the mice, provide mechanistic insights into the *CDSN*-nEDD. Nevertheless, grafting skin from *Cdsn^epi−/−^* mouse in wt mouse allows for the studying of the effects of the absence of Cdsn in the long-term. Such study has revealed the infiltration of inflammatory cells in the dermis [6]. In this direction, grafting of HEE in mice could also allow investigating the interplay between epidermis with the underlying dermis and with immune cells. Another potentially useful strategy to study the absence of Cdsn in the long-term, and at the same time allow testing of potential therapeutics (e.g., small molecules, protein replacement, etc.) could involve the generation of a mosaic *Cdsn^−/−^* mouse that could overcome the neonatal lethality as in the case with the *SPINK5*-sEDD [41].

In conclusion, the data presented here indicates that elimination of KLK5 does not rescue the *CDSN*-nEDD phenotype. On the contrary, KLK5 ablation likely aggravates over-desquamation associated with a severe skin barrier defect in this case. The study advances the understanding of the mechanisms that govern the *CDSN*-nEDD pathology.

## 4. Materials and Methods

### 4.1. Materials

All chemicals used were obtained from Sigma-Aldrich (Louis, MO, USA). The fluorescent substrates were obtained from Thermo Scientific, Waltham, MA, USA. The antibodies were obtained from commercial sources as follow: Keratin 5, Abcam (ab24647), Cambridge, UK; Dsg1, Santa Cruz (sc-20114), Santa Cruz, CA, USA; loricrin, Abcam (ab24722); involucrin, Santa Cruz (sc-15230) and Sigma (I908), St. Louis, MO, USA; Dsc1, Santa Cruz (sc-18115), KLK5 (R&D Systems, AF2008, Minneapolis, MN, USA), and actin (Chemicon, MAB1501, Tokyo, Japan). The anti-corneodesmosin monoclonal antibody G36-19 was produced and characterized in our laboratory [25].

### 4.2. Human Samples

Human abdominal skin samples from healthy donors undergoing plastic surgery were obtained from Genoskin (Toulouse, France), following their written informed consent, as authorized by the French Ministry of Research (#AC-2017-2897). Tape strippings from lesional and non-lesional skin area of a *CDSN*-mutated patient were collected for diagnosis and gathered in a biological collection (n°DC-2011-1388, French National Ethics Committees). Written informed consent was obtained from the patient’s legal representatives.

### 4.3. Mice

*Klk5^−/−^* mice generated previously are viable, fertile, and macroscopically identical to wild-type (wt) mice [13]. *Cdsn^epi−/−^* are epidermal conditional corneodesmosin knockout mice that have been generated by K14-promoter driven Cre-mediated deletion of *Cdsn* gene in the epidermis [6]. All mice are of the C57BL6 background. Genotyping was conducted as described previously [6,13]. All experiments with animals were approved by the local Ethics Committee and carried out according to our institutional guidelines and European Union legislation. Specifically, all experiments with animals were approved by the General Directorate of Rural Economy and Veterinary Matters (Region of Western Greece) with number 187537/627 on 26 June 2018.

### 4.4. Cell Culture and Generation of HEE

Primary normal human epidermal keratinocytes (NHEK) culture and production of HEE were performed as previously described [42]. NHEsK were established from human abdominal skin samples and grown in Dermalife culture medium (CellSystems, Troisdorf, Germany). 350,000 subconfluent keratinocytes in suspension in EpiLife medium containing 1.5 mmol/L calcium were seeded on polycarbonate culture inserts (area of 0.63 cm^2^ with pores 0.4 mm in diameter; Merck Millipore, Molsheim, France). After 48 hours of incubation at 37 °C in a humidified atmosphere containing 5% CO_2_, cells were exposed to the air–liquid interface (which corresponded to day zero of differentiation), and 50 mg/mL vitamin C (Sigma-Aldrich, Louis, MO, USA) and 10 ng/mL keratinocyte growth factor (Sigma-Aldrich) were added to the medium in the lower compartment. The medium was renewed every two days until harvesting on Day 12 of the air-exposed phase.

### 4.5. shRNA Lentiviral Particles and Keratinocyte Transduction

We proceeded as previously described [42]. In brief, we designed different shRNA sequences for each target gene (*CDSN* and *KLK5*) and the lentiviral particles were produced in pLKO.1 vectors (Table S1), with puromycin (in the case of sh*CDSN*) or neomycin (in the case of sh*KLK5*) antibiotic resistance gene. Gene silencing efficiency was assessed for each shRNA vs. control (nontarget shRNA) lentiviral particles and two shRNA per target gene were selected for further experiments. For the double silencing experiments, one pair of lentiviral particles targeting *CDSN* and *KLK5*, respectively, was chosen for co-transduction of keratinocytes. Each single- or double-silencing experiment was repeated using keratinocytes from two different donors.

### 4.6. In Situ Zymography

In situ zymography to detect epidermal proteolytic activities was conducted on 5 μm skin cryosections, as described previously [26]. Briefly, skin cryosections were incubated in 2% Tween-20 in PBS for 2 min followed by a wash in PBS and then, incubated with 10 μg/mL BODIPY FL casein, 10 μg/mL BODIPY FL elastin or 10 μg/mL BODIPY FL gelatin substrates at 37 °C. Then, the excess substrate was removed, and biopsies were covered with Mowiol medium and observed under a confocal laser scanning electron microscope (Leica TCS SP5, Leica Biosystems, Nussloch, Germany).

### 4.7. Western Blot

HEEs were lyzed in Laemmli sample buffer and processed for Western blot analysis using the primary antibodies, as previously described [42]. The detection was realized with the ECL Prime system (GE Healthcare, Chicago, IL, USA) and images were acquired with a G:BOX Chemi XT4 CCD camera (Syngene, Cambridge, UK) and GeneSys software (version 1.0.7.154, Genesys, Daly City, CA, USA). ImageJ software (version 1.52a, National Institutes of Health, Bethesda, MD, USA) was used to quantify immunoreactive bands. Signals were normalized to actin immunodetection.

### 4.8. Transmission Electron Microscopy (TEM)

Skin biopsies from newborn mice of all genotypes (i.e., wt, *Cdsn^−/−^, Klk5^−/−^* and DKO *Cdsn^epi−/−^Klk5^−/−^*) or from HEEs were collected, fixed in 2% glutaraldehyde and 4% formaldehyde in 0.1 M cacodylate buffer, then, washed, stained with 1% OsO_4_, dehydrated in a series of acetone baths and, finally, embedded in epoxy-resin (Sigma-Aldrich). Ultrathin sections were mounted on 100 mesh collodion-coated copper grids and post-stained with 3% uranyl acetate and 8.5% lead citrate before being examined with a Hitachi HT7700 electron microscope (Hitachi, Tokyo, Japan).

### 4.9. Scanning Electron Microscopy (SEM)

Neonates were decapitated and the heads were fixed in 4% formaldehyde in PBS for 24 h, and then, dehydrated in a series of ethanol solutions 25, 50 and 100% for 10 min. Ethanol was replaced with 100% acetone by two washes for 10 min each. The samples were allowed to dry completely, gold sputtered and observed in a field emission scanning electron microscope (JEOL, Pleasanton, CA, USA, 6300).

### 4.10. Toluidine Blue Penetration and Dehydration Assay

The staining and the loss of water of newborn mice were carried out as described previously [13].

### 4.11. Lucifer Yellow Permeability Assay

The assay was performed as previously described [42]. Dye concentration in the culture medium was measured fluorometrically in a Fluoroskan Ascent (Thermo Scientific, Waltham, MA, USA) with excitation and emission at 425 and 550 nm, respectively.

### 4.12. Histology and Immunohistochemistry (IHC)

Skin from mice or HEE were fixed with 4% paraformaldehyde in PBS for 24 h. Then, the tissues were embedded in paraffin, cut to 5 μm sections with a microtome and processed for hemalun-eosin staining.

IHC was performed in 5 μm skin cryosections which were fixed in acetone for 10 min and then, were air dried at room temperature for 5 min, followed by hydration in PBS for 5 min. Endogenous peroxidase activity was blocked by incubation in 3% H_2_O_2_ in PBS for 10 min and blocking of unspecific binding-sites was conducted by incubation with 0.3% BSA in 0.1% Triton X-100 in PBS for 5 min. Slides were then, incubated with the primary antibodies either for 1h at room temperature or overnight in a humidity chamber at 4 °C depending on the working conditions of each antibody. Then, secondary HRP-conjugated antibody in 0.3% BSA in PBS was applied in section for 30 min at room temperature. After this, slides were washed twice (5 min per step) with 0.3% BSA in PBS and 1 time with PBS for 5 min. Finally, the slides were incubated with DAB substrate for 10 min and counterstained with hematoxylin.

### 4.13. Lipid Extraction and ^1^H-NMR

Lipids were extracted from the epidermis of wt, *Cdsn^−/−^*, *Klk5^−/−^*, and *Cdsn^−/−^Klk5^−/−^* mice with chloroform/methanol/PBS (2:1:0.1). The lipids were normalized against the protein content. The solvents were evaporated, and the lipid extracts were dissolved in CDCl_3_ for ^1^H-NMR analysis. A known concentration of methanol was added as an internal standard to assist in quantification. From human samples, lipids were extracted with the same procedure but from the SC. ^1^H-NMR (available at the University of Patras, Center for Instrumental Analysis) spectra were recorded at 22 ± 1 °C on a Bruker AM-600 WB spectrometer (Billerica, MA, USA) operating at a field strength of 600 MHz for ^1^H. Peak assignment was based on Kriat et al., [43], Adosraku et al., [44], and Tynkkynen [45].

### 4.14. Stratum Corneum pH Measurement

The measurement of pH at the surface of the HEE was carried out using the pH meter HI 99181 (Hanna Instrument, Smithfield, RI, USA) equipped with the electrode HI 1414D/50.

### 4.15. Oil Red Staining of HEE Cryosections

Cryosections (5 µm-thick) from HEE previously embedded in Tissue-Tek OCT compound (VWR, Radnor, PA, USA) were fixed with 4% formaldehyde-containing buffer. After a brief rinse in phosphate-buffered saline and 30% isopropanol, sections were stained with freshly prepared oil red O (Sigma-Aldrich) working solution for 15 min at room temperature, rinsed in 30% isopropanol and distilled water, and mounted in ProLong Gold Antifade Mountant containing 4,6-diamidino-2-phenylindole (DAPI, Invitrogen Life Technologies, Carlsbad, CA, USA). Images were captured using a ×63 magnification oil immersion lens in a Zeiss Apotome microscope (Carl Zeiss, Jena, Germany).

### 4.16. Cornified Envelopes Maturation Assay

Cornified envelopes were isolated from HEE samples as previously described [46]. Appropriate concentrations of cornified envelope suspension were dropped onto a slide-glass and air-dried. They were fixed in acetone at −20 °C for 10 min, and hydrated in phosphate-buffered saline. The primary antibody anti-Involucrin (SY5) allowed a reaction for 1 h at room temperature. A secondary antibody was then applied for 1 h at room temperature. After washing, the cornified envelopes were stained with Nile red solution (1 mg/mL) for 30 min at room temperature. Slides were then mounted in Mowiol. Images were taken by fluorescence microscopy using a Nikon eclipse 80i microscope equipped with a Nikon DXM 1200C digital camera (Tokyo, Japan) and NIS image analysis software (v. 5.21.00).

### 4.17. RNA Extraction and RT-qPCR

Total RNA was extracted with the Nucleospin RNA kit (Macherey-Nagel, Düren, Germany). One μg of total RNA was reversed-transcribed with Superscript First-Strand Synthesis System for RT-PCR (Invitrogen). For quantitative PCR, primer sequences were designed using Primer3 software to generate amplicons of 150–200 bp. BLAST+ 2.8.1 analysis ensured the absence of similarity to any other human sequence. The primers used are listed in Table S2.

## Data Availability

All data are included in the manuscript and the Supplementary Materials.

## Supplementary Materials

The following supporting information can be downloaded at: https://www.mdpi.com/article/10.3390/ijms26178605/s1.

## Abbreviations

The following abbreviations are used in this manuscript:

CDSN	corneodesmosin
DKO	double knockout
EDD	epidermal differentiation disorder
HEE	human epidermal equivalent
SC	*stratum corneum*
SG	*stratum granulosum*
TEM	transmission electron microscopy
