# Supplementary material for: Deletion of the Epidermal Protease KLK5 Aggravates the Symptoms of Congenital Ichthyosis CDSN-nEDD
Source: Int J Mol Sci. 2025 Sep 4;26(17):8605. doi: 10.3390/ijms26178605 (PMC12429418; doi:10.3390/ijms26178605)
Supplement: Supplementary file 1 [file ijms-26-08605-s001.zip › ijms-3829345-supplementary.pdf]

Deletion of the epidermal protease *KLK5* aggravates the symptoms of the congenital ichthyosis *CDSN*-nEDD

Supplementary Materials

(Supplementary Figures S1-S3, and Table S1, S2)

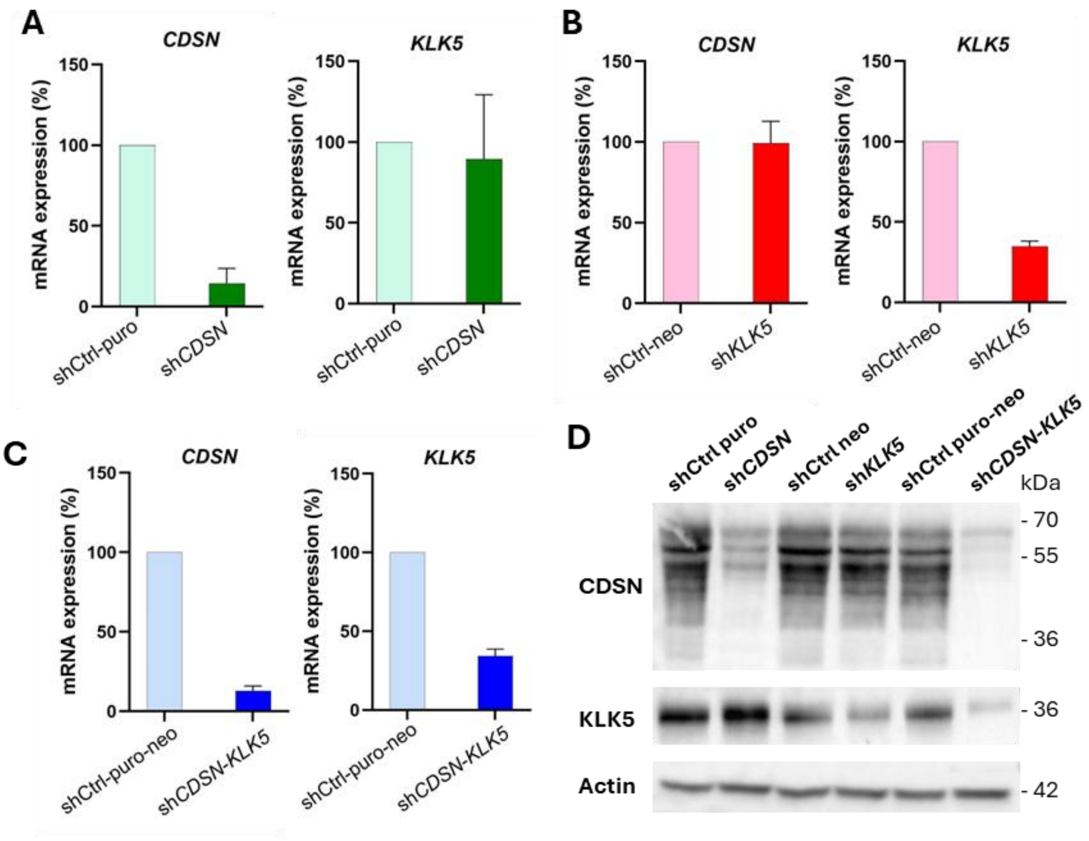

Figure S1. Expression of *CDSN* and/or *KLK5* is highly reduced in HEEs produced with sh*CDSN* and/or sh*KLK5* keratinocytes. Fully differentiated HEEs were produced after transduction with control shRNA (shCtrl puro and/or shCtrl neo), sh*CDSN* and/or sh*KLK5*. (**A-C**) The resulting HEEs were analyzed by RT-qPCR for *CDSN* and *KLK5* gene expression, as indicated. The results for a given HEE are presented as a percentage relative to the corresponding control HEE. (**D**) Protein extracts from the resulting HEEs were analyzed by western blotting using anti-CDSN or anti-KLK5 antibodies, as indicated. Protein loading was verified with anti-actin antibody.

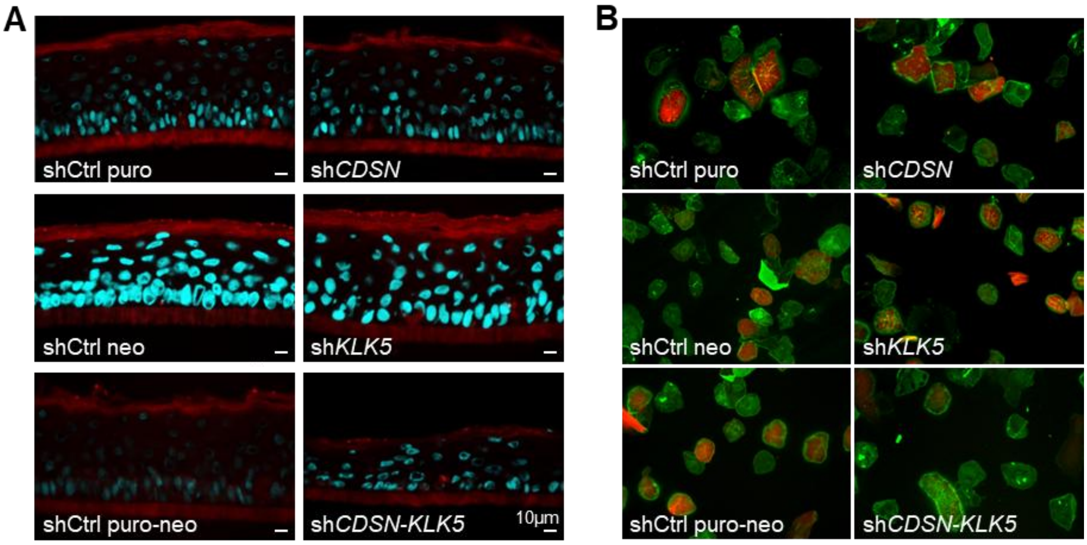

Figure S2: Effect of CDSN and/or KLK5 gene silencing on the SC lipids and maturation of cornified envelopes. (A) Oil Red O staining of lipids on HEE frozen sections. (B) Double staining of cornified envelopes purified from the indicated HEE using Nile Red (red) and an antibody specific to involucrin (green). n ≥ 2 independent experiments for each condition.

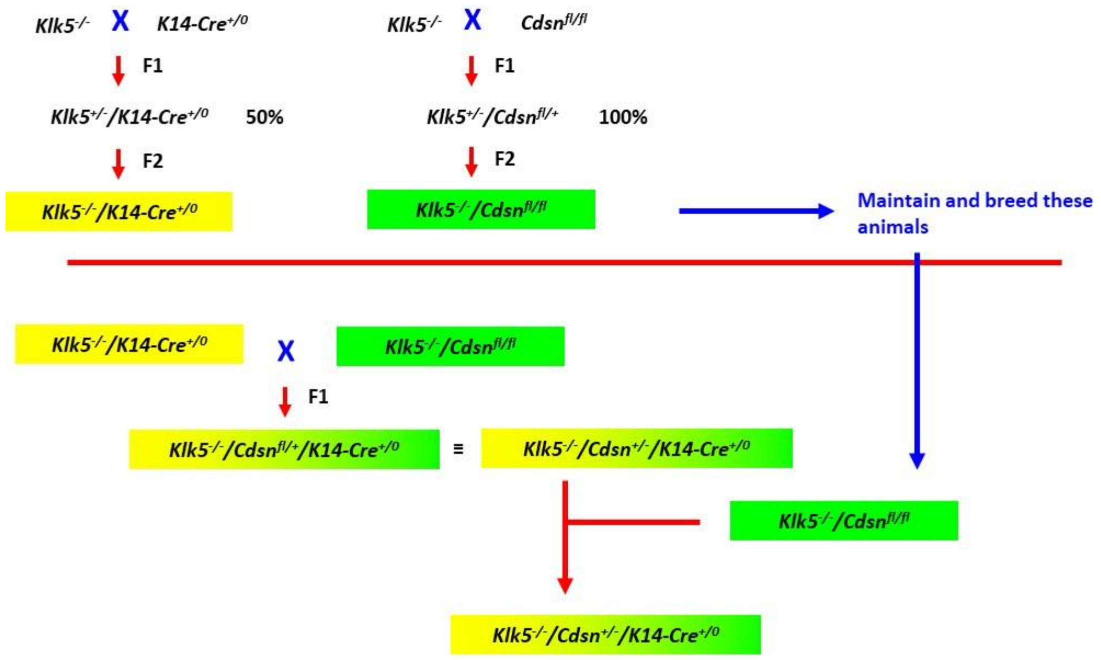

Figure S3. Schematic diagram of the breeding strategy used here.

Table S1: shRNA lentiviral vectors used in the study

Vector	Clone ID	DNA sequence	Target RNA
pLKO.1-Neo	TRCN0000073997	CCCTGTCACCAGTTTATGAAT	*KLK5* (NM_012427)
pLKO.1-Neo	TRCN0000378888	CATCAACGTCTCCTCTCATTG	*KLK5* (NM_012427)
pLKO.1-Neo	TRCN0000372710	ACTCCTTTCAGACCCTCATTC	*KLK5* (NM_012427)
pLKO.1-Neo	TRCN0000372709*	CAATGGATCCGACTGCGATAT	*KLK5* (NM_012427)
pLKO.1-Puro	TRCN0000083130	GCTCTAGTCTACAAGGTGCAT	*CDSN* (NM_001264)
pLKO.1-Puro	TRCN0000083132*	TCCAGGCATGACCTACAGTAA	*CDSN* (NM_001264)
pLKO.1-Puro	SHC002V	GCAACAAGATGAAGAGCACCAA	Non-Mammalian shRNA Control
pLKO.1-Neo	CSTVRS	GCAACAAGATGAAGAGCACCAA	Non-Mammalian shRNA Control

**shRNA selected for the double transduction experiments*

Table S2: List of PCR primers used in the study

Name	Sequence (5'-3')
CDSN_F	ACTGCTGCTGGCTGGTCT
CDSN_R	AGAGCTTCTGGCACTGGAAA
KLK5_F	AGTCAGAAAAGGTGCGAGGA
KLK5_R	TGAACTTGCAGAGGTTCGTG
YWHAZ_F	ACTTTTGGTACATTGTGGCTTCAA
YWHAZ_R	CCGCCAGGACAAACCAGTAT
